# 2-(1*H*-Benzimidazol-2-yl)phenol

**DOI:** 10.1107/S1600536814001366

**Published:** 2014-01-22

**Authors:** S. M. Prakash, A. Thiruvalluvar, S. Rosepriya, N. Srinivasan

**Affiliations:** a Research and Development Center, Bharathiar University, Coimbatore 641 046, Tamilnadu, India; b Department of Chemistry, Annamaliar College of Engineering, Mudaiyur 606 902, Tamilnadu, India; c Postgraduate Research Department of Physics, Rajah Serfoji Government College (Autonomous), Thanjavur 613 005, Tamilnadu, India; d Department of Chemistry, S.K.P. Engineering College, Thiruvannamalai 606 611, Tamilnadu, India

## Abstract

The title mol­ecule, C_13_H_10_N_2_O, is essentially planar, the maximum deviation from the plane of the non-H atoms being 0.016 (2) Å. The imidazole ring makes a dihedral angle of 0.37 (13)° with the attached benzene ring. An intra­molecular O—H⋯N hydrogen bond generates an *S*(6) ring motif. In the crystal, mol­ecules are linked through N—H⋯O hydrogen bonds, forming chains propagating in [001]. The crystal packing also features four π–π stacking inter­actions involving the imidazole ring, fused benzene ring and attached benzene ring system [centroid–centroid distances = 3.6106 (17), 3.6108 (17), 3.6666 (17) and 3.6668 (17) Å].

## Related literature   

For applications and general background to substituted benzimidazole derivatives, see: Nakamura *et al.* (2004 ▶); Su Han & Kim (2001 ▶); Roman *et al.* 2007 ▶; Congiu *et al.* 2008 ▶. For related crystal structures, see: Han (2010 ▶); Zhan *et al.* (2007 ▶). For hydrogen-bond motifs, see: Bernstein *et al.* (1995 ▶). For bond-length data, see: Allen *et al.* (1987 ▶). *Note added in proof*: a low temperature determination of the same structure has been reported [Konoshima, H., Nagao, S., Kiyota, I., Amimoto, K., Yamamoto, N., Sekine, M., Nakata, M., Furukawa, K. & Sekiya, H. (2012). *Phys. Chem. Chem. Phys.* **14**, 16448–16457].
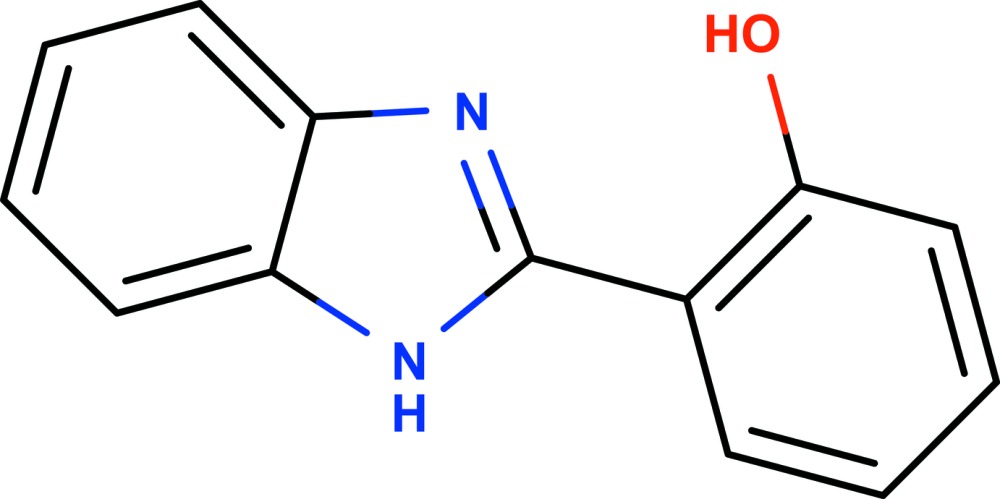


## Experimental   

### 

#### Crystal data  

C_13_H_10_N_2_O
*M* *_r_* = 210.23
Monoclinic, 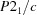

*a* = 16.864 (4) Å
*b* = 4.7431 (8) Å
*c* = 12.952 (2) Å
β = 102.34 (2)°
*V* = 1012.1 (3) Å^3^
*Z* = 4
Mo *K*α radiation
μ = 0.09 mm^−1^
*T* = 293 K
0.30 × 0.30 × 0.25 mm

#### Data collection  

Agilent Xcalibur Eos Gemini diffractometer
Absorption correction: multi-scan (*CrysAlis PRO*; Agilent, 2013 ▶) *T*_min_ = 0.829, *T*_max_ = 1.000
4073 measured reflections
2338 independent reflections
1184 reflections with *I* > 2σ(*I*)
*R*_int_ = 0.037

#### Refinement  

*R*[*F* ^2^ > 2σ(*F* ^2^)] = 0.067
*wR*(*F* ^2^) = 0.131
*S* = 1.03
2338 reflections
150 parameters
H atoms treated by a mixture of independent and constrained refinement
Δρ_max_ = 0.18 e Å^−3^
Δρ_min_ = −0.17 e Å^−3^

### 

Data collection: *CrysAlis PRO* (Agilent, 2013 ▶); cell refinement: *CrysAlis PRO*; data reduction: *CrysAlis PRO*; program(s) used to solve structure: *SIR2011* (Burla *et al.*, 2012 ▶); program(s) used to refine structure: *SHELXL2013* (Sheldrick, 2008 ▶); molecular graphics: *ORTEP-3 for Windows* (Farrugia, 2012 ▶) and *PLATON* (Spek, 2009 ▶); software used to prepare material for publication: *SHELXL2013* and *PLATON*.

## Supplementary Material

Crystal structure: contains datablock(s) global, I. DOI: 10.1107/S1600536814001366/hg5377sup1.cif

Structure factors: contains datablock(s) I. DOI: 10.1107/S1600536814001366/hg5377Isup2.hkl

Supporting information file. DOI: 10.1107/S1600536814001366/hg5377Isup3.cdx

Supporting information file. DOI: 10.1107/S1600536814001366/hg5377Isup4.cml

CCDC reference:

Additional supporting information:  crystallographic information; 3D view; checkCIF report

## Figures and Tables

**Table 1:** Hydrogen-bond geometry (Å, °)

*D*—H⋯*A*	*D*—H	H⋯*A*	*D*⋯*A*	*D*—H⋯*A*
N1—H1⋯O26^i^	0.91 (2)	1.96 (3)	2.851 (3)	169 (2)
O26—H26⋯N3	0.82	1.81	2.551 (3)	150

Symmetry code: (i) 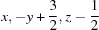.

**Fig. 1.**
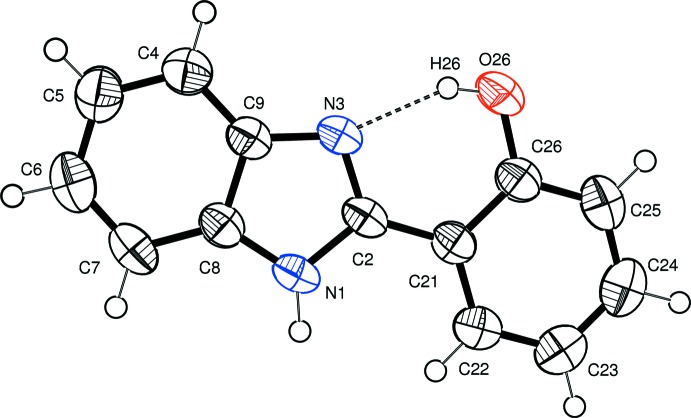
The molecular structure of the title compound, with displacement ellipsoids drawn at the 50% probability level. H atoms are shown as small spheres of arbitrary radius. The dashed line indicates the intramolecular O—H···N hydrogen bond.

**Fig. 2.**
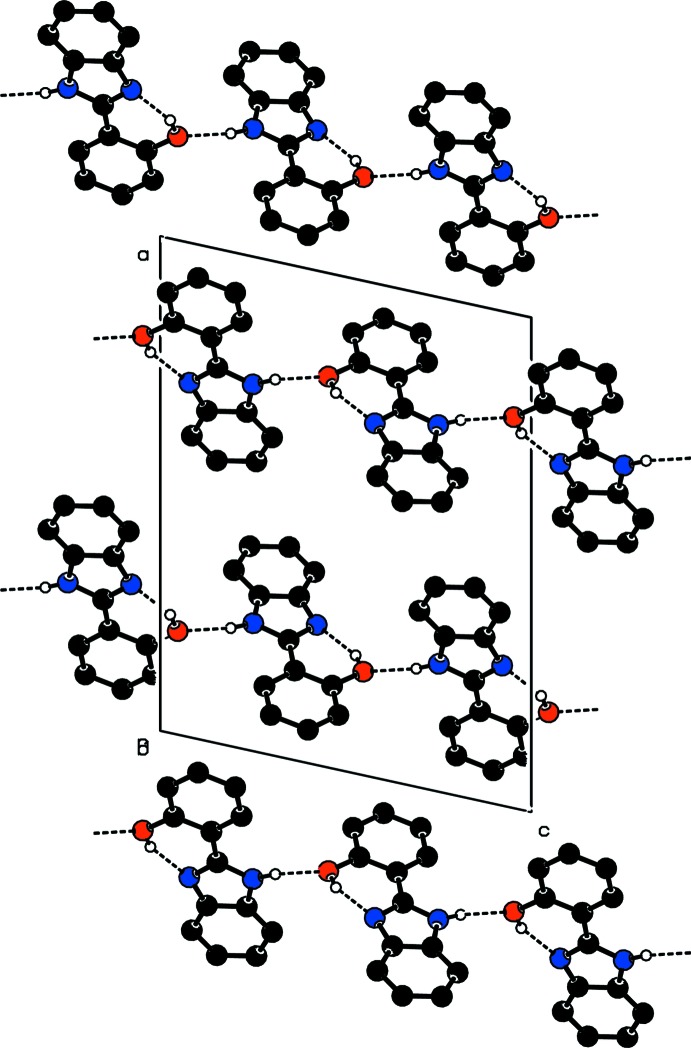
The packing of the title compound, viewed down the b axis. Dashed lines indicate hydrogen bonds. H atoms not involved in hydrogen bonding have been omitted.

**Fig. 3.**
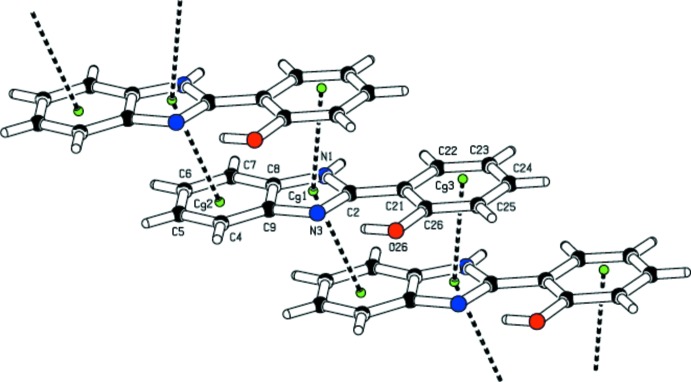
Part of the crystal structure of compound, showing the formation of π-π stacking interactions.

## supplementary crystallographic information

### 1. Comment

Imidazole derivatives have occupied a unique place in the field of medicinal chemistry. Many of the substituted imidazoles are known as inhibitors of P38 map kinase, fungicides and herbicides and therapeutic agents (Nakamura *et al.*, 2004; Su Han & Kim, 2001). Being a polar and ionisable aromatic compounds, it improves pharmacokinetic characteristics of lead molecules and thus used as a remedy to optimize solubility and bioavailability parameters of proposed poorly soluble lead molecules. The imidazole ring is a constituent of several important natural products, including purine, histamine, histidine and nucleic acid (Roman *et al.*, 2007; Congiu *et al.*, 2008). Owing to the wide range of pharmacological and biological activities, the synthesis of imidazoles has become an important target in current years. We are interested to study the biological and photo physical properties of 2-(1*H*-benzimidazol-2-yl)phenol. The related compounds whose structures have been solved by X-ray are 2-(1*H*-Benzimidazol-2-yl)-4,6-dichlorophenol (Han, 2010) and 4-(1*H*-Benzo[*d*]imidazol-2-yl)phenol (Zhan *et al.*, 2007). As part of our research, we have synthesized the title compound and report its crystal structure here.

The title molecule, C_13_H_10_N_2_O, (Fig. 1), is essentially planar, the maximum deviation from the plane of the non-H atoms being 0.016 (2) Å for O26. The imidazole ring (N1/C2/N3/C9/C8) makes dihedral angle of 0.37 (13)° with the attached benzene ring (C21—C26).

An intramolecular O26—H26···N3 hydrogen bond generates an S(6) ring motif (Bernstein *et al.*, 1995). In the crystal, symmetry-related molecules are linked through N1—H1···O26 hydrogen bonds, forming an one dimensional chains propagating in [001] (Fig. 2).

The crystal packing also features four π-π stacking interactions. [*Cg*1—*Cg*2^i^ = 3.6668 (17) Å, *Cg*1—*Cg*3^ii^ = 3.6106 (17) Å, *Cg*2—*Cg*1^ii^ = 3.6666 (17) Å and *Cg*3—*Cg*1^i^ = 3.6108 (17) Å, symmetry codes (i): *x*, 1 + *y*, *z*; (ii): *x*, -1 + *y*, *z*. Where, *Cg*1 is the centroid of the imidazole ring (N1/C2/N3/C9/C8), *Cg*2 is the centroid of the fused benzene ring (C4—C9) and *Cg*3 is the centroid of the attached benzene ring (C21—C26) respectively] (Fig. 3). The N—C, C═N, C_ar_—C_ar_ and C—O bond lengths in (I) are within their normal ranges (Allen *et al.*, 1987).

### 2. Experimental

To the pure *o*-phenylenediamine (1.6 g, 15 mmol) in ethanol (10 ml), 2-hydroxybenzaldehyde (1.6 g, 15 mmol) and ammonium acetate (3 g) was added about 1 h by maintaining the temperature at 353 K. The reaction mixture was refluxed for 48 hrs and extracted with dichloromethane. The solid separated was purified by column chromatography using benzene as the eluent. Yield: 1.89 g; 60%. The compound was dissolved in benzene and ethyl acetate (9:1) mixture and allowed to slow evaporation for two days, to obtain crystals suitable for X-ray diffraction studies.

### 3. Refinement

The N-bound H atom was located in a difference Fourier map and refined freely; N1—H1 = 0.91 (2) Å. The remaining H atoms were positioned geometrically and allowed to ride on their parent atoms, with O—H = 0.82 and C*sp*^2^—H = 0.93 Å. *U*_iso_(H) = *1.5U*_eq_(O) and *1.2U*_eq_(C).

### Crystal data

**Table:**

C_13_H_10_N_2_O	*F*(000) = 440
*M**_r_* = 210.23	*D*_x_ = 1.380 Mg m^−^^3^
Monoclinic, *P*2_1_/*c*	Melting point: 386 K
Hall symbol: -P 2ybc	Mo *K*α radiation, λ = 0.71073 Å
*a* = 16.864 (4) Å	Cell parameters from 612 reflections
*b* = 4.7431 (8) Å	θ = 4.8–23.8°
*c* = 12.952 (2) Å	µ = 0.09 mm^−^^1^
β = 102.34 (2)°	*T* = 293 K
*V* = 1012.1 (3) Å^3^	Block, colourless
*Z* = 4	0.30 × 0.30 × 0.25 mm

### Data collection

**Table:**

Agilent Xcalibur Eos Gemini diffractometer	2338 independent reflections
Radiation source: Enhance (Mo) X-ray Source	1184 reflections with *I* > 2σ(*I*)
Graphite monochromator	*R*_int_ = 0.037
Detector resolution: 16.3291 pixels mm^-1^	θ_max_ = 29.2°, θ_min_ = 3.2°
ω scans	*h* = −23→10
Absorption correction: multi-scan (*CrysAlis PRO*; Agilent, 2013)	*k* = −3→6
*T*_min_ = 0.829, *T*_max_ = 1.000	*l* = −15→17
4073 measured reflections	

### Refinement

**Table:**

Refinement on *F*^2^	Primary atom site location: structure-invariant direct methods
Least-squares matrix: full	Secondary atom site location: difference Fourier map
*R*[*F*^2^ > 2σ(*F*^2^)] = 0.067	Hydrogen site location: mixed
*wR*(*F*^2^) = 0.131	H atoms treated by a mixture of independent and constrained refinement
*S* = 1.03	*w* = 1/[σ^2^(*F*_o_^2^) + (0.0339*P*)^2^] where *P* = (*F*_o_^2^ + 2*F*_c_^2^)/3
2338 reflections	(Δ/σ)_max_ < 0.001
150 parameters	Δρ_max_ = 0.18 e Å^−^^3^
0 restraints	Δρ_min_ = −0.17 e Å^−^^3^

### Special details

**Table:**

Geometry. Bond distances, angles *etc*. have been calculated using the rounded fractional coordinates. All su's are estimated from the variances of the (full) variance-covariance matrix. The cell e.s.d.'s are taken into account in the estimation of distances, angles and torsion angles

### Fractional atomic coordinates and isotropic or equivalent isotropic displacement parameters (Å^2^)

**Table:**

	*x*	*y*	*z*	*U*_iso_*/*U*_eq_	
O26	0.20929 (12)	0.7018 (4)	0.54707 (12)	0.0640 (8)	
N1	0.25872 (13)	0.5474 (4)	0.25071 (16)	0.0459 (8)	
N3	0.28328 (12)	0.4482 (4)	0.42170 (14)	0.0448 (7)	
C2	0.24046 (15)	0.6055 (5)	0.34606 (18)	0.0420 (8)	
C4	0.38923 (16)	0.0807 (5)	0.4149 (2)	0.0561 (10)	
C5	0.42862 (16)	−0.0543 (5)	0.3475 (2)	0.0603 (11)	
C6	0.41275 (17)	0.0093 (6)	0.2410 (2)	0.0621 (11)	
C7	0.35740 (17)	0.2089 (5)	0.1985 (2)	0.0554 (10)	
C8	0.31718 (15)	0.3444 (5)	0.26679 (19)	0.0438 (9)	
C9	0.33257 (15)	0.2825 (5)	0.37415 (18)	0.0434 (9)	
C21	0.18120 (15)	0.8077 (4)	0.36215 (19)	0.0424 (8)	
C22	0.13653 (15)	0.9671 (5)	0.28084 (19)	0.0512 (9)	
C23	0.08097 (17)	1.1585 (5)	0.2980 (2)	0.0614 (11)	
C24	0.06758 (18)	1.1945 (5)	0.3980 (3)	0.0640 (11)	
C25	0.11064 (18)	1.0406 (5)	0.4800 (2)	0.0617 (11)	
C26	0.16741 (16)	0.8493 (5)	0.4634 (2)	0.0485 (9)	
H1	0.2390 (15)	0.643 (5)	0.190 (2)	0.068 (9)*	
H4	0.40039	0.03737	0.48660	0.0673*	
H5	0.46693	−0.19179	0.37380	0.0722*	
H6	0.44063	−0.08687	0.19714	0.0748*	
H7	0.34708	0.25216	0.12684	0.0666*	
H22	0.14474	0.94254	0.21266	0.0613*	
H23	0.05206	1.26504	0.24222	0.0737*	
H24	0.02909	1.32393	0.40988	0.0768*	
H25	0.10138	1.06568	0.54762	0.0741*	
H26	0.23899	0.58738	0.52659	0.0960*	

### Atomic displacement parameters (Å^2^)

**Table:**

	*U*^11^	*U*^22^	*U*^33^	*U*^12^	*U*^13^	*U*^23^
O26	0.0835 (16)	0.0762 (13)	0.0334 (11)	0.0152 (10)	0.0147 (10)	−0.0050 (9)
N1	0.0540 (15)	0.0546 (13)	0.0295 (12)	−0.0037 (11)	0.0100 (10)	0.0028 (11)
N3	0.0511 (14)	0.0521 (12)	0.0304 (11)	0.0005 (11)	0.0070 (10)	−0.0010 (10)
C2	0.0486 (16)	0.0465 (14)	0.0314 (14)	−0.0094 (12)	0.0095 (12)	−0.0032 (12)
C4	0.0567 (19)	0.0620 (17)	0.0475 (17)	0.0017 (14)	0.0068 (14)	−0.0017 (14)
C5	0.0532 (18)	0.0608 (17)	0.067 (2)	0.0028 (14)	0.0129 (15)	−0.0089 (16)
C6	0.062 (2)	0.0648 (17)	0.067 (2)	−0.0066 (16)	0.0304 (16)	−0.0132 (16)
C7	0.067 (2)	0.0610 (17)	0.0436 (17)	−0.0111 (16)	0.0241 (15)	−0.0073 (14)
C8	0.0454 (16)	0.0462 (14)	0.0410 (15)	−0.0080 (13)	0.0122 (12)	−0.0022 (12)
C9	0.0443 (16)	0.0488 (14)	0.0370 (15)	−0.0055 (13)	0.0087 (12)	−0.0015 (12)
C21	0.0454 (16)	0.0424 (13)	0.0391 (15)	−0.0070 (12)	0.0087 (12)	−0.0033 (12)
C22	0.0537 (17)	0.0547 (15)	0.0440 (17)	−0.0030 (14)	0.0081 (13)	0.0020 (13)
C23	0.0558 (19)	0.0583 (17)	0.067 (2)	0.0025 (15)	0.0062 (16)	0.0056 (15)
C24	0.0547 (19)	0.0539 (16)	0.085 (2)	0.0048 (14)	0.0187 (17)	−0.0042 (17)
C25	0.067 (2)	0.0645 (18)	0.059 (2)	0.0004 (16)	0.0255 (16)	−0.0112 (15)
C26	0.0534 (18)	0.0508 (15)	0.0410 (16)	−0.0033 (13)	0.0092 (13)	−0.0051 (13)

### Geometric parameters (Å, º)

**Table:**

O26—C26	1.355 (3)	C21—C26	1.394 (4)
O26—H26	0.8200	C21—C22	1.382 (3)
N1—C2	1.363 (3)	C22—C23	1.357 (4)
N1—C8	1.362 (3)	C23—C24	1.372 (5)
N3—C9	1.381 (3)	C24—C25	1.363 (4)
N3—C2	1.317 (3)	C25—C26	1.369 (4)
N1—H1	0.91 (2)	C4—H4	0.9300
C2—C21	1.432 (3)	C5—H5	0.9300
C4—C9	1.376 (4)	C6—H6	0.9300
C4—C5	1.364 (4)	C7—H7	0.9300
C5—C6	1.381 (4)	C22—H22	0.9300
C6—C7	1.361 (4)	C23—H23	0.9300
C7—C8	1.383 (4)	C24—H24	0.9300
C8—C9	1.390 (3)	C25—H25	0.9300
			
C26—O26—H26	109.00	C22—C23—C24	119.8 (2)
C2—N1—C8	107.5 (2)	C23—C24—C25	120.1 (3)
C2—N3—C9	106.09 (19)	C24—C25—C26	120.4 (3)
C8—N1—H1	127.3 (16)	O26—C26—C21	121.1 (2)
C2—N1—H1	124.9 (16)	O26—C26—C25	118.6 (2)
N1—C2—N3	111.4 (2)	C21—C26—C25	120.3 (2)
N1—C2—C21	124.5 (2)	C5—C4—H4	121.00
N3—C2—C21	124.0 (2)	C9—C4—H4	121.00
C5—C4—C9	118.3 (2)	C4—C5—H5	119.00
C4—C5—C6	121.3 (2)	C6—C5—H5	119.00
C5—C6—C7	121.7 (3)	C5—C6—H6	119.00
C6—C7—C8	116.9 (2)	C7—C6—H6	119.00
N1—C8—C7	132.0 (2)	C6—C7—H7	122.00
C7—C8—C9	122.0 (2)	C8—C7—H7	122.00
N1—C8—C9	106.1 (2)	C21—C22—H22	119.00
C4—C9—C8	119.8 (2)	C23—C22—H22	119.00
N3—C9—C8	108.9 (2)	C22—C23—H23	120.00
N3—C9—C4	131.3 (2)	C24—C23—H23	120.00
C2—C21—C22	122.6 (2)	C23—C24—H24	120.00
C2—C21—C26	119.6 (2)	C25—C24—H24	120.00
C22—C21—C26	117.8 (2)	C24—C25—H25	120.00
C21—C22—C23	121.6 (2)	C26—C25—H25	120.00
			
C8—N1—C2—N3	−0.8 (3)	C6—C7—C8—N1	−179.1 (3)
C8—N1—C2—C21	−179.7 (2)	C6—C7—C8—C9	0.5 (4)
C2—N1—C8—C7	180.0 (3)	N1—C8—C9—N3	0.1 (3)
C2—N1—C8—C9	0.4 (3)	N1—C8—C9—C4	179.5 (2)
C9—N3—C2—N1	0.8 (3)	C7—C8—C9—N3	−179.6 (2)
C9—N3—C2—C21	179.7 (2)	C7—C8—C9—C4	−0.1 (4)
C2—N3—C9—C4	−179.9 (3)	C2—C21—C22—C23	−179.7 (2)
C2—N3—C9—C8	−0.6 (3)	C26—C21—C22—C23	0.1 (4)
N1—C2—C21—C22	−0.4 (4)	C2—C21—C26—O26	0.1 (4)
N1—C2—C21—C26	179.9 (2)	C2—C21—C26—C25	−179.6 (2)
N3—C2—C21—C22	−179.2 (2)	C22—C21—C26—O26	−179.6 (2)
N3—C2—C21—C26	1.1 (4)	C22—C21—C26—C25	0.7 (4)
C9—C4—C5—C6	0.3 (4)	C21—C22—C23—C24	−0.7 (4)
C5—C4—C9—N3	179.0 (3)	C22—C23—C24—C25	0.7 (4)
C5—C4—C9—C8	−0.3 (4)	C23—C24—C25—C26	0.0 (4)
C4—C5—C6—C7	0.1 (4)	C24—C25—C26—O26	179.6 (2)
C5—C6—C7—C8	−0.4 (4)	C24—C25—C26—C21	−0.7 (4)

### Hydrogen-bond geometry (Å, º)

**Table:**

*D*—H···*A*	*D*—H	H···*A*	*D*···*A*	*D*—H···*A*
N1—H1···O26^i^	0.91 (2)	1.96 (3)	2.851 (3)	169 (2)
O26—H26···N3	0.82	1.81	2.551 (3)	150

Symmetry code: (i) *x*, −*y*+3/2, *z*−1/2.
